# Efficiency of drone technology for lake water sampling: is it better than traditional boat methodology?

**DOI:** 10.1007/s10661-026-15473-0

**Published:** 2026-05-22

**Authors:** Juan Federico Bennett, Brian Rippey, Richard Douglas

**Affiliations:** School of Geography and Environmental Sciences, Ulster University, Cromore Rd, Coleraine, BT52 1SA UK

**Keywords:** Uncrewed aerial vehicles, Water monitoring, Costs, Ireland

## Abstract

Water pollution is one of the main socio-environmental issues in recent decades, affecting humans and various species. The increasing use of drones can be an alternative that helps more easily monitor the status of hard-to-reach water bodies, making their examination more accurate and allowing for the implementation of appropriate restoration measures. In this study, we investigated the use of drones to monitor three small shallow lakes in Ireland, an island with hundreds of lakes. The objective of this study is to compare the drone sampling methodology versus the traditional boat-based methodology, evaluating aspects such as the reliability of the results and cost-effectiveness. Key physicochemical water parameters such as total phosphorus, chlorophyll a, dissolved oxygen and pH were measured over a three-month period using multiparameter probes and water sample collection. Both methodologies yielded identical results for nine of the ten measured variables. In turn, the drone method, while entailing higher initial fixed costs, ends up being not only faster but also more economical in the medium and long term, also representing a safer and more far-reaching sampling alternative than the boat methodology.

**Supplementary Information:**

The online version contains supplementary material available at 10.1007/s10661-026-15473-0.

## Introduction

Water pollution due to industrial and agronomic activities is one of the main causes of pathologies and death on our planet, with children being a particularly vulnerable group and a large amount of flora and fauna also being affected (Ore et al., 2015; Singh et al., 2021; Zhang et al., 2023). Uncrewed aerial vehicles (UAVs), usually known as drones, have acquired greater relevance in recent decades due to their use in varied fields, including lake, river or coastal water sampling (Graham et al., 2022; Lally et al., 2019; Shelare et al., 2021). A frequent and simpler sampling of lake water is indispensable for the analysis of key variables such as chlorophyll *a*, electrical conductivity, turbidity, algal densities and different kinds of contaminants such as persistent organic pollutants (Farinha et al., 2022; Powers et al., 2018; Ruiz-Villarreal et al., 2022).

Although many lakes or aquatic ecosystems have a walkable shoreline and jetty, an advantage of drone sampling is the ability to work on formerly inaccessible areas that may have fencing, dense tree cover, muddy shorelines, unavailability for van parking and boat deployment or intrinsically complex attributes, such as in mangroves, estuaries, coral reefs, volcanoes and arctic lakes (Doi et al., 2017; Hodgson et al., 2022; Neto et al., 2022; Terada et al., 2018; Vélez-Nicolás et al., 2021). Furthermore, working with drones enables sampling hazardous lakes such as quarries or mining reservoirs (Banerjee et al., 2020; Benson et al., 2019; Koparan et al., 2018a; Shelare et al., 2021).

Ireland is an optimal location for the use of drones in lake water sampling since the island has hundreds of shallow lakes that are not assessed by the EPA (Irish Environmental Protection Agency) or selected as a European Union Water Framework Directive (WFD) monitoring priority due to their small surface area, non-use for drinking water supply, safety issues and difficulty deploying boats (Lally et al., 2020). Many of these water bodies are in the surface range between 1 and 100 ha, and simpler sampling would allow more lakes to be included in improved and more frequent monitoring, leading to better management decisions (Gibson & Jordan, 2002). Graham et al. (2022) also explained that large-scale monitoring programmes such as the WFD or the United Nation Global Environment Monitoring System for Freshwater (GEMS/water) would benefit from intensive and standardised use of drones for routine monitoring.

This study presents results from Corcaghan, Greagh and Grove lakes in which, for 3 months, simultaneous water sampling by traditional boat method and assisted by drone was carried out. The planning, execution and outcomes of this work serve as a starting point to compare both methods. Differences in the measured variables between both methodologies, a cost and time efficiency analysis and other key points such as technical features of the sampling devices, will help elucidate whether the drone method is an advance worth implementing for lake water sampling.

Hypotheses

- Water physicochemical variables measured with traditional boat methodology return the same results as when measured by the drone methodology.
- Remote freshwater sampling with drone technology represents a valuable alternative that can replace or complement standard boat sampling.

## Materials and methods

### Study site

**Fig. 1:**
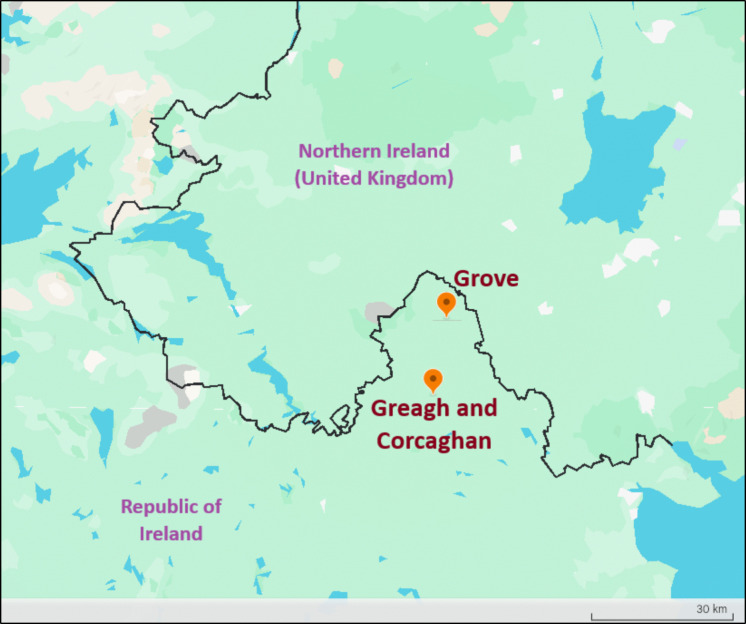
Location of the three selected lakes, in the Republic of Ireland, close to the border with Northern Ireland (UK) (source: Google Earth)

The research was performed in three small rural lakes (Fig. 1) in County Monaghan, Republic of Ireland: Corcaghan (54.19° N, 7.01° W; area, 4 ha; mean depth, 3 m), Greagh (54.18° N, 7.02° W; area, 3 ha; mean depth, 3.5 m) and Grove (54.32° N, 6.96° W; area, 1 ha; mean depth, 3 m).

### UAV and sampling system features

The used DJI Matrice 300 RTK quadcopter weighs 6.3 kg (with batteries). The drone has a maximum payload capacity of 2.7 kg, making it suitable for attaching a YSI EXO 1 sonde, which weighs 1.42 kg; or a 500-ml LDPE bottle of water with a ballast (Fig. 3), which altogether weighs 1.93 kg. The aircraft supports a flight time between 31 and 37 min with the used payload (55 min maximum without payload) and has a wind resistance of 15 m/s and a –20 to 50 ℃ operating temperature range (DJI, 2023). The model is suitable for long field work activities with light or medium rain (DJI Enterprise, 2020).

### Drone-based versus traditional sampling

**Fig. 2:**
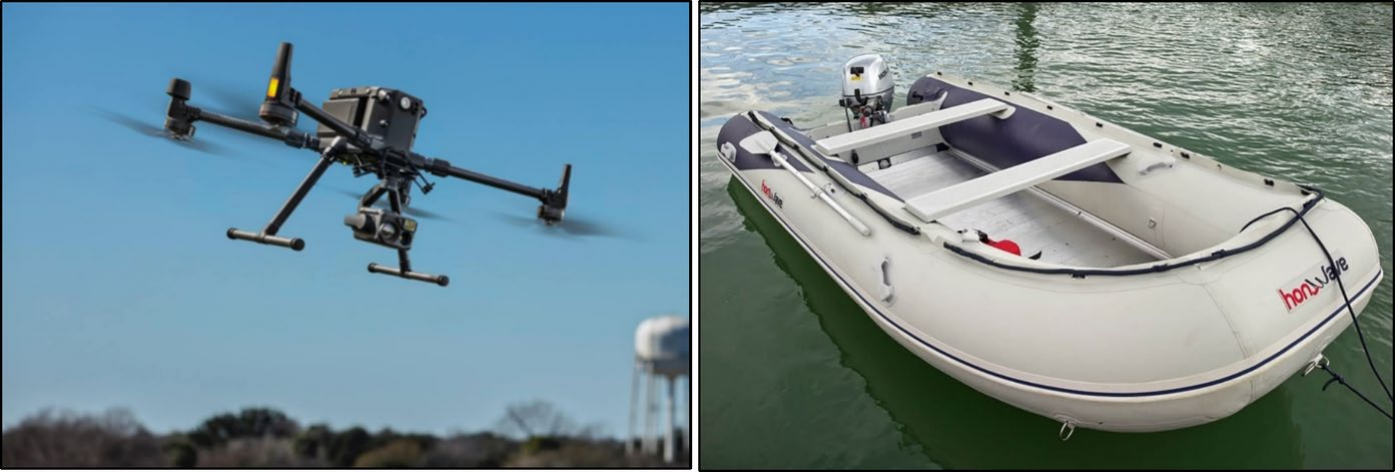
DJI Matrice 300 RTK drone and Honwave T24IE3 inflatable boat (images taken from DJI, 2023 and Honda, 2024)

From August to October 2023, Corcaghan, Greagh and Grove lakes’ water parameters were analysed with a YSI EXO 1 sonde and in the laboratory, after water collection with a LDPE 500-ml bottle. Both devices were attached one after another to a DJI Matrice 300 RTK commercial C3 drone by a 5-m-long nylon rope. The lakes’ water was also monitored using a Ruttner bottle and the YSI EXO 1, from a boat (Fig. 2). Water samples were retrieved for analysing alkalinity, pH, total aluminium, total phosphorus, chlorophyll *a*, total iron and total manganese. The sonde measured dissolved oxygen, specific conductivity and turbidity, in addition to other general variables such as water temperature and depth.

For drone operation, our team and institution (Ulster University) possessed valid flyer and operator IDs, respectively, and the corresponding coverage liability (insurance) for the UK and the Republic of Ireland. Training and examinations had been completed under UK Civil Aviation Authority (CAA) regulations for categories A1 and A3, and all regulations required by the CAA and the IAA (Irish Aviation Authority) were met.

**Fig. 3:**
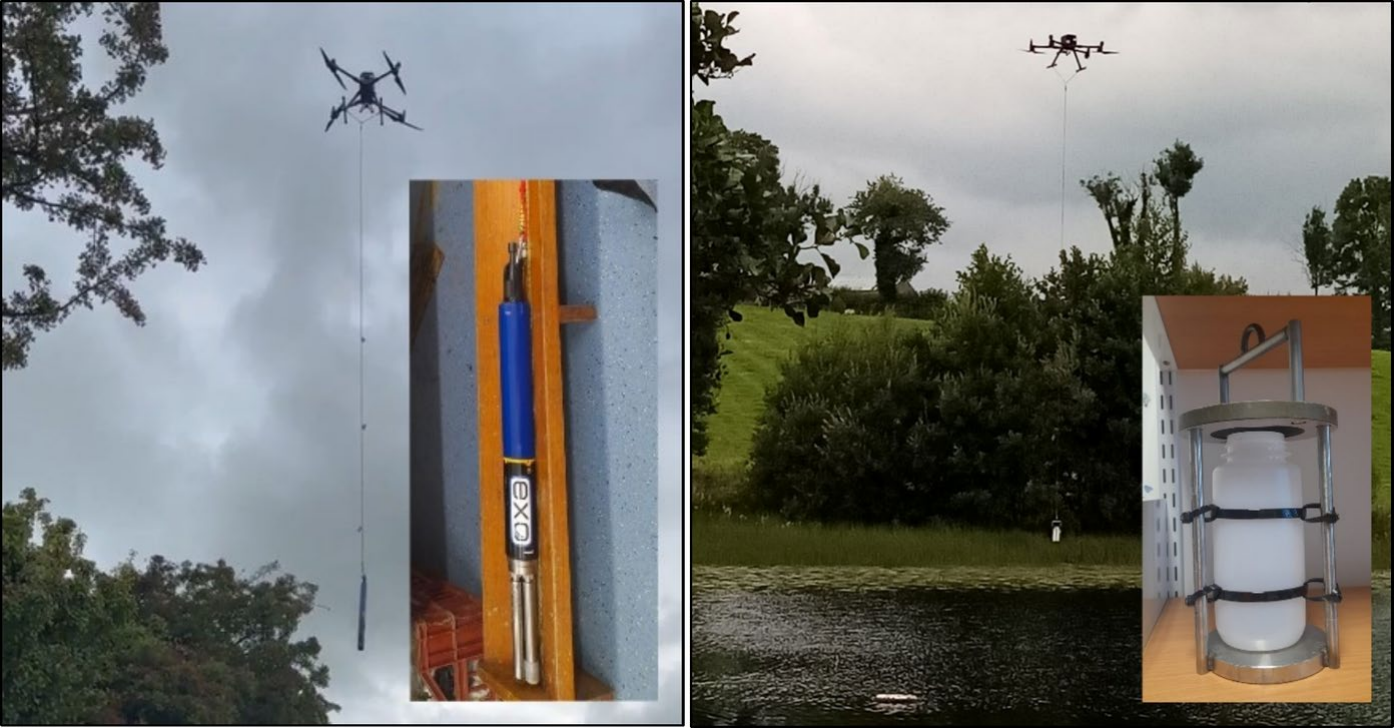
On the left**,** the YSI EXO 1 sonde hanging from the drone (using the 5 m rope) and a close-up image of the sonde. On the right, the 500-ml LDPE water bottle hanging from the drone before being submerged in the lake (attached to a metal ballast for adequate immersion). A close-up image of the device is also shown

Three days (August 30, September 21 and October 12, 2023) were selected to test the sampling methodologies in the studied lakes. A central point of each lake was selected to take the sonde and water samples for both modalities. First, the drone was flown to that point (about 100 m from the shore and within visual range) and then slowly descended. The rope-attached YSI EXO 1 (Fig. 3) was immersed in the water to measure the variables at 1, 2 and 3 m depth. The YSI EXO 1 was left at least for 20 s at each depth to allow the measured physicochemical variables stabilise, and to discern representative values for each depth, the data was then downloaded and displayed in a spreadsheet. The sensors recorded data every second, providing an optimal solution for the assessment of profile depths. Subsequently, the drone was retrieved to the shore, the YSI EXO 1 was replaced by the 500 ml bottle and ballast and the drone was returned to the centre of the lake but this time to collect a subsurface water sample. The ballast was added to reduce wind disturbance and effectively immerse the bottle in the water.

Each of the two flights on each lake was performed with a pair of batteries, and the power provided was sufficient to complete the mission, with the battery charge dropping to 50–55%. When beginning the same procedures on the second lake, the pair of batteries was swapped, and the same was executed for the third lake, finally using the six available batteries.

Subsequently, the boat-based measurements were taken, after navigating with an inflatable boat towards the same central point. The boat was anchored to fix the position. With the YSI EXO sonde, the same parameters were measured at 1, 2 and 3 m depths, and afterwards, the water sample was retrieved with the Ruttner bottle and stored in prewashed plastic bottles.

### Depth measurements

During the drone procedures, the depth to which the sonde was submerged was measured indirectly using the real-time flight altitude reported by the drone’s smart controller. For instance, if the rope length was of 5 m and we wanted to measure at 3 m depth, the corresponding drone altitude to be shown in the Smart controller was of 2 m. On the other hand, to measure the depth of the water from the boat, using the YSI sonde or the Ruttner sampler, the cables of the devices were marked every metre with a yellow tape, allowing to know the exact location of the 1, 2 and 3 m of depth. For Ruttner’s water samples, a messenger included in the equipment was used, which could be released when the desired depth was reached, and this mechanism locked the bottle so that it could be recovered with the water of that depth.

### Downloading data from the YSI EXO 1 to a laptop

The drone was used to transfer the YSI EXO 1 from a take-off point near the shore to a central point of the lakes (about 100 m from the shore). The sonde was connected via bluetooth to a laptop. When the sonde is taking readings close to the computer, live data of all the selected variables can be visualised in real time in the Kor software - v1.5.0.223. When the signal is lost due to the long distance between the two devices, a function allows the sonde to continue recording the data, although the live information cannot be displayed. Once the sonde is recovered, the recorded data can be downloaded.

### EXO 1 probes and water samples analysis

The approximate measurement uncertainty of dissolved oxygen, specific conductivity and turbidity was 1%, 1% and 3% of the readings, respectively (YSI, 2024). Total aluminium (± 3%), total phosphorus (± 5%), chlorophyll a (± 20%), iron (± 2%) and manganese (± 15%) absorbances were determined by UV–Vis spectrophotometry in a Shimadzu UVmini 1240 and UV-1800 spectrophotometer after adding colorimetric reagents. Employing a Metrohm 916 Ti Touch titrator, pH (± 5%) and alkalinity (± 3%) were determined by titrating unfiltered water samples with HCl to the equivalence point, defined primarily by the pK value of the first ionisation of carbonic acid, around pH 4.5 (APHA, 1985).

### Cost-efficiency analysis

The time employed to collect water samples using boat and drone methodologies in small, shallow lakes was estimated, including unpacking and packing of equipment. The costs of sampling were determined, and the main reference for this estimate was the purchased items and technology described, along with the cost of standard labour wages.

### Data analysis

Repeated measures tests (also known as matched tests) were selected as it was necessary to compare the overall variability between sampling methodologies (boat and drone) for each measured variable considering pairs of results and not a general average of all replicates. For example, dissolved oxygen concentration in September for 3 m depth in Corcaghan for boat methodology was compared against the same variable concentration in September for 3 m depth in Corcaghan for drone methodology. The use of paired samples eliminated the noise from the natural variability of the results between different lakes, months or depths.

Parametric tests were performed since the distribution of the measured variables was normal, their variances were homogeneous and the compared groups had an identical number of repetitions. Since two groups were compared, repeated measures *t*-test was chosen. A significance level of *p* ≤ 0.05 was selected. The IBM SPSS Statistics software 28.0 was employed to execute the tests.

Additional data on Materials and Methods are given in the Supplementary Material (Online Resource 1).

## Results

Comparing the boat and drone methodologies to monitor lake water with a sonde (Table 1), all dissolved oxygen (Test: repeated measures *t*-test, *n* = 27, *p* = 0.43), specific conductivity (Test: repeated measures *t*-test, *n* = 27, *p* = 0.08) and turbidity (Test: repeated measures *t*-test, *n* = 27, *p* = 0.17) showed no significant differences considering all months, lakes and depths globally.

**Table 1:** Mean values of dissolved oxygen, specific conductivity and turbidity for Corcaghan, Greagh and Grove, on sampling days of August, September and October 2023, for boat and drone sampling methodologies using the YSI EXO 1. The average of all the values (one-second frequency readings for at least 20 s) was calculated for each depth (1, 2 and 3 m)

Variables	Depth (m)	Lakes	Boat			Drone			*p*-value
			Aug	Sep	Oct	Aug	Sep	Oct	
Dissolved oxygen (%)	1	Corcaghan	76.5	86.2	73.2	74.9	85.9	78.2	0.43
		Greagh	89.4	96.1	74.0	94.9	95.0	87.6	
		Grove	77.0	67.1	64.1	75.7	75.1	58.8	
	2	Corcaghan	68.0	83.3	67.5	65.6	82.0	68.6	
		Greagh	86.5	92.0	69.4	86.9	90.7	70.7	
		Grove	61.3	50.5	47.3	44.6	45.7	39.6	
	3	Corcaghan	63.2	74.4	62.6	58.7	77.0	65.8	
		Greagh	84.6	91.6	68.0	83.9	85.9	67.0	
		Grove	11.0	39.2	31.8	23.3	18.1	25.0	
Specific cond. (µS/cm)	1	Corcaghan	181.9	182.3	180.8	183.2	183.9	181.5	0.08
		Greagh	134.8	140.6	140.7	138.4	144.3	141.9	
		Grove	370.5	371.1	385.6	373.8	365.3	387.1	
	2	Corcaghan	181.8	182.4	179.7	183.2	183.8	181.8	
		Greagh	136.7	143.1	140.7	138.4	144.5	141.8	
		Grove	371.1	373.4	385.8	384.9	377.0	392.1	
	3	Corcaghan	181.8	183.9	180.1	183.4	184.6	180.7	
		Greagh	138.9	143.4	140.9	138.6	144.7	142.1	
		Grove	429.0	372.5	391.7	426.2	461.8	433.7	
Turbidity (FNU)	1	Corcaghan	6.0	4.9	4.5	6.6	5.4	5.1	0.17
		Greagh	9.8	3.9	3.1	9.7	4.0	3.4	
		Grove	1.0	1.9	1.3	1.5	2.2	1.7	
	2	Corcaghan	6.2	5.2	4.4	7.3	6.0	4.7	
		Greagh	9.3	4.0	3.1	9.6	4.1	3.4	
		Grove	0.7	1.9	1.1	2.5	1.9	1.0	
	3	Corcaghan	7.7	5.1	4.1	127.1	8.4	11.5	
		Greagh	9.3	4.0	3.0	9.6	3.7	3.4	
		Grove	4.6	0.5	1.3	7.0	11.4	16.3	

Nevertheless, there are specific cases where the differences between methodologies are large, such as turbidity values for some lakes and months, especially at 3 m depth. This is due to the intrinsic heterogeneity of turbidity. The sensor used works by shining a near-infrared light beam and measuring the light scattered by suspended particles. Lake water is not a homogeneous medium, and suspended particles of different size and colour can abruptly alter the values of the variable, even in a matter of seconds (YSI, 2024). This pattern is more detectable at 3 m depth, due to the resuspension of sediment if the sonde accidentally hits it or bioturbation caused by fish (Inland Fisheries Ireland, 2025). Thus, the measured turbidity is likely to vary from second to second.

**C**omparing the boat and drone methodologies to monitor lake water with a sonde (Table 2), chlorophyll a (Test: repeated measures *t*-test, *n* = 9, *p* = 0.33), total phosphorus (Test: repeated measures *t*-test, *n* = 9, *p* = 0.69), iron (Test: repeated measures *t*-test, *n* = 9, *p* = 0.48), manganese (Test: repeated measures *t*-test, *n* = 9, *p* = 0.54), aluminium (Test: repeated measures *t*-test, *n* = 9, *p* = 0.59) and alkalinity (Test: repeated measures *t*-test, *n* = 9, *p* = 0.24) showed no significant differences considering all months and lakes globally. Only pH showed significant differences considering all months and lakes (Test: repeated measures *t*-test, *n* = 9, *p* = 0.01).

**Table 2:** Mean values of aluminium, alkalinity, pH, total phosphorus, chlorophyll *a*, iron and manganese for Corcaghan, Greagh and Corcaghan, on sampling days of August, September and October 2023, for boat and drone sampling methodologies. For both methods, a single subsurface water sample was collected with a 0.5 L bottle of water/ Ruttner bottle, and the value obtained from its analysis in the laboratory for each variable is taken as the mean

Variables	Lakes	Boat			Drone			*p*-value
		Aug	Sep	Oct	Aug	Sep	Oct	
Aluminium (μg/l)	Corcaghan	56.4	68.7	59.6	59.3	68.5	59.3	0.59
	Greagh	38.8	37.4	30.1	37.6	36.6	31.6	
	Grove	11.6	9.8	10.1	11.8	9.6	10.3	
Alkalinity (meq/l)	Corcaghan	1.5	1.6	1.5	1.6	1.6	1.6	0.24
	Greagh	1.2	1.3	1.2	1.2	1.3	1.2	
	Grove	3.4	3.3	3.5	3.3	3.3	3.5	
pH	Corcaghan	7.1	6.9	7.0	7.5	7.5	7.4	0.01
	Greagh	6.9	6.9	6.9	7.0	7.0	6.9	
	Grove	7.6	7.3	7.5	7.7	7.7	7.7	
Total Phosphorus (μg/l)	Corcaghan	191.6	146.9	152.8	196.4	145.6	155.1	0.69
	Greagh	77.1	87.9	65.1	90.3	88.4	63.8	
	Grove	51.3	72.5	34.5	41.5	61.9	47.2	
Chlorophyll a (μg/l)	Corcaghan	17.9	20.8	33.7	20.8	35.8	25.1	0.33
	Greagh	31.5	56.6	85.3	73.8	119.0	63.1	
	Grove	21.5	46.6	21.5	10.8	42.3	28.0	
Iron (μg/l)	Corcaghan	1237.8	1477.6	1257.3	1244.3	1467.9	1241.1	0.48
	Greagh	1030.5	1114.7	829.6	1024.0	1046.7	858.7	
	Grove	86.7	45.7	63.2	75.8	54.0	69.9	
Manganese (μg/l)	Corcaghan	487.1	445.0	374.1	492.3	459.5	367.5	0.54
	Greagh	184.2	445.7	380.6	131.0	437.8	385.2	
	Grove	58.0	54.1	100.7	60.0	54.1	104.7	

The duration of water sampling employing the traditional boat methodology is 45 min, while for the drone-based approach is 28 min, the former being 60% more time consuming for a single lake sample (Table 3).

**Table 3:** Estimated capital costs of water sampling for the boat and drone-based methodologies (including VAT). Light blue font indicates items that are identical for both methodologies and add the same amount of capital to the final amount. The presented values consider an initial purchase cost of materials and do not include equipment repair costs or the costs related to arrival at the lakes, which should be similar between methods. It is assumed that the useful life and maintenance costs are similar for the two techniques. Green font indicates items related to labour utilisation time and the times required for each task are shown. The costs consider a standard worker salary of 0.33 US dollars per minute for the drone methodology and 0.67 for the boat methodology, since the latter requires two workers for safety reasons related to working on the water. For the elements in green, the sampling costs for 710 lake samples are shown as an example

**Item or task**	Cost type	Task duration for one lake (boat) in minutes	Task duration for one lake (drone) in minutes	Boat-based water sampling duration for 710 lakes (minutes)	Drone-based water sampling duration for 710 lakes (minutes)	Costs of boat-based water sampling (USD)	Costs of drone-based water sampling (USD)
Honwave T24IE3 inflatable boat	Fixed (initial)					1364.00	
Mariner 4-Stroke 3.5hp Outboard Engine						1080.00	
Oars (2) and anchor						165.00	
DJI Matrice 300 RTK Drone and Remote Smart Controller							10,625.00
TB 60 Flight batteries (6)							5378.00
DJI Matrice 300 RTK Battery station							1269.00
YSI EXO 1 Multiparameter Sonde						6803.00	6803.00
YSI probes						6418.00	6418.00
Water sampler						945.00	945.00
Boat/drone setup	Variable	20	10	14,200.00	7100.00	9466.67	2366.67
Boat/drone sampling		15	11	10,650.00	7810.00	7100.00	2603.33
Boat/drone disassembly		10	7	7100.00	4970.00	4733.33	1656.67
		45	28	31,950.00	19,880.00	**38,075.00**	**38,064.67**

**Fig. 4:**
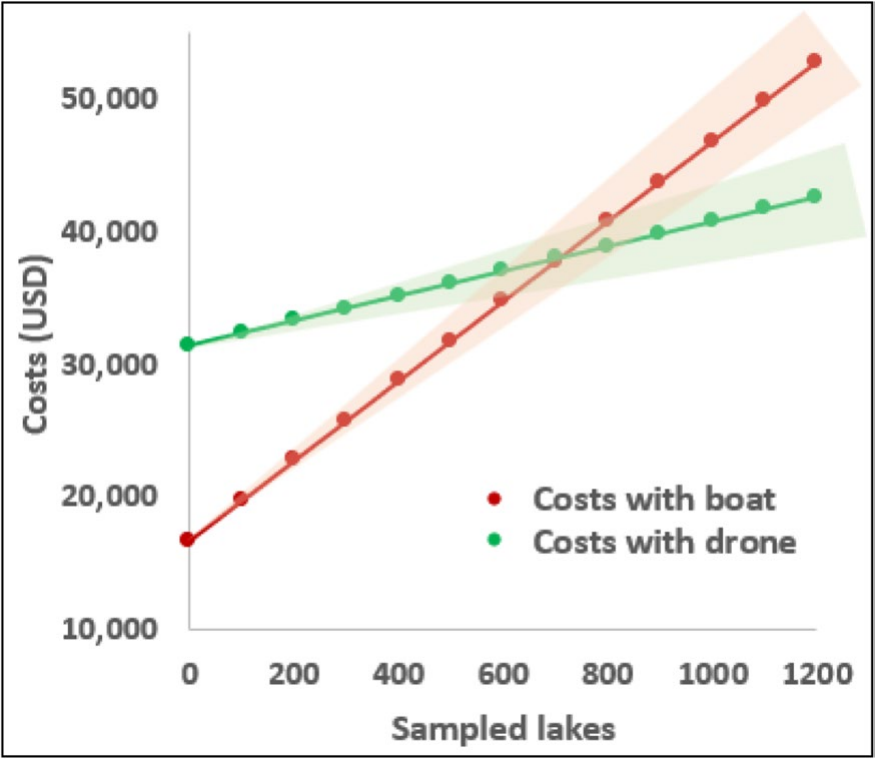
Capital costs of lake sampling using the boat methodology and the drone methodology in relation to the number of lake samples. The translucent area surrounding both lines represents different scenarios generated by considering different unit prices for a variable cost, such as workers' wages. In turn, changes in fixed costs would modify the y-intercept of the functions

The overall initial fixed cost of water sampling employing the traditional boat methodology is USD 16,775, while for a drone-based approach, the value is USD 31,438, the latter being 88% more expensive (Table 3). However, the variable costs associated with human labour and duration of sampling tasks are lower for the drone methodology. The cost of the boat methodology exceeds that of the drone after 710 samples (Fig. 4). That number may seem elevated, but any research group at a university or environmental government agency that primarily focuses on sampling water bodies for follow-up can typically collect hundreds of water samples per month. This means that 710 is a threshold achievable in just a few months, not years.

## Discussion

### Data comparison between boat and drone methodologies

Many investigations that studied water sampling with the use of UAVs did not apply statistical tests to their differences with the traditional boat method, while others were able to do so. For instance, Koparan et al. (2018b) collected three replicate water samples of each sampling spot of a shallow lake. They obtained small but not significant percentage differences for some variables such as dissolved oxygen, which was 3.6%. According to the authors, the discrepancy was due to the error range of the used dissolved oxygen metre, which was 0.2 mg/L.

Detweiler et al. (2015) explain that the differences in dissolved oxygen may be due to the time lag between collection and analysis, since this variable fluctuates rapidly in a matter of hours due to possible extended degassing during transport to the laboratory. Furthermore, diurnal variation can cause differences in dissolved oxygen of up to 3 mg/L, so non-synchronic measurements can be a source of uncertainty. In our experiment, all variables were measured with the sonde or by laboratory analysis of collected water samples, but not simultaneously with both methods, so this source of differences in the results was ruled out.

On the other hand, Castendyk et al. (2020) measured the relative percentage difference (RPD) between the reported values of each variable for both methodologies. They obtained RPDs less than 20% for almost all measured variables, which is the threshold defined by the US EPA to meet data quality of chemical data for duplicates and consider them equivalent (US EPA, 1994).

With respect to our results (Tables 1 and 2), there were some variables such as chlorophyll a that showed considerable differences between methodologies in analogous measures (same lake, month and depth), but the statistical differences were not significant for the entire variable (*P* = 0.33). This occurred because the methodology that yielded higher values was not always the same for different lakes, months and depths, with the overall mean for each methodology being almost identical. To demonstrate this, Table 2 shows that for chlorophyll a in August, the drone methodology yielded greater values at Corcaghan (20.8 µg/l vs 17.9 µg/l in boat methods), and the boat methodology yielded greater values at Grove (21.5 µg/l vs 10.8 µg/l in the drone method).

The opposite pattern took place in variables such as pH, where the relative difference between analogous samples taken by the different methodologies was short, but it was the drone methodology that consistently showed greater values than the boat methodology, making the differences for the entire variable significant (*P* = 0.01). Graham et al. (2022) found no significant differences between boat and drone methodologies except for pH (as in our case). In their study, they applied repeated measures* t*-tests or also a Wilcoxon ranked test calculating a coefficient of variation and the average of each variable.

A potential explanation for the moderately higher pH values using the drone method at Corcaghan Lake is that photosynthesis occurred in the collected water during the minutes it took for the water sample to travel from the sampling point to the shore. The collection bottle used in the drone method, unlike the Ruttner bottle, was uncapped and more transparent, favouring photosynthesis, a process that raises the pH of the water. Due to its larger size and specific shape, the water collection point at Corcaghan was farther away than at the other two lakes, and the maximum altitude reached by the drone after water retrieval was higher due to a large curtain of trees that had to be avoided between the shore and the landing point. All of this resulted in a considerably longer recovery landing than at the other two lakes (Graham et al., 2022; Koparan et al., 2018b). However, although the differences were statistically significant, they were small enough not to have ecological or physicochemical implications.

In any case, pH was the only variable out of the ten evaluated (3 measured with sondes and 7 by laboratory analysis of water samples) that showed significant differences between the methods. These results prove the first hypothesis of the article, showing that the drone and boat methods are consistent and generate very similar results.

### Cost-efficiency analysis

The main reason for the higher initial fixed cost of the drone methodology (Table 3) is the value of the DJI Matrice 300 RTK drone (USD 10625, including VAT) compared to the cost of the Honwave T24IE3 inflatable boat (USD 1364). However, these prices may vary depending on the brands and models selected. Vélez-Nicolás et al. (2021) explain that lightweight drones typically used for water sampling have extremely variable prices from USD 675 up to USD 135,000.

Zhang et al. (2023) explained that the boat sampling requires two operators, one driving the boat and the other one for sampling, while a drone only needs one person to complete the associated tasks. There is little danger associated with single-person work with a drone, as there are no safety risks related to working directly on the water surface, such as drowning.

The main time saving in the drone method compared to the boat method is that the setup (and also the disassembly) of the drone takes half the time, not involving the lifting of heavy weights and tedious steps such as removing the boat from the boot of the van, deploying it on the lake and inflating it manually. The sampling is also faster with the drone methodology as it does not involve launching the boat from the shore, traveling to the centre of the lake and anchoring the boat.

In their studied lake in Ireland, Lally et al. (2020) conducted a cost-efficiency analysis. Considering different scenarios and whether or not handheld probes were used, their drone-based water sampling was between 130 and 240% faster than with the traditional method and also between 17 and 63% more expensive. Our results agree that the drone methodology initial fixed costs are greater than those of the boat but at the same time is considerably faster and has lower variable costs, suggesting that a regular use for a few years or even just months can justify the higher initial investment of money in pursuit of greater long-term efficiency.

Regarding the deterioration of components, the propellers are a sensitive part of the drone, and their replacement frequency is relevant for optimal performance. If cared for and inspected regularly, propellers can typically be used for 300 h or 3 months if used permanently (DJI, 2023; Dronesgator, 2024). If used occasionally, they can be used for many months or years. DJI Enterprise (2020) suggests that regular drone maintenance should be performed every 3–6 months, but they consider daily use. Additionally, DJI 300 Matrice RTK purchase provides an array of spare propellers, so component replacement is not necessarily more frequent compared to the boat, which also experiences material deterioration and frequent damage to components, especially in sensitive parts such as the engine or propeller.

### Payload weight relevance

Graham et al. (2022) extracted 2-L replicate samples with both boat and drone methodologies for their 12 studied lakes. These authors affirmed that larger drones allow bigger payloads and help have less biased samples that come from higher quantities of water. Shelare et al. (2021) warned that pH determination may be biased by drone water collection due to the small amount of water that can generally be collected and may prevent a representative measurement of the real value. For instance, a minimum of 50 ml of sample was necessary to conduct the pH determination in Corcaghan, Greagh and Grove, as recommended by the Standard Methods for the Examination of Water and Wastewater (APHA, 1985). However, volumes currently extracted by drones that are usually reported in the literature (Table 4) are sufficient to analyse the main variables and elements. These procedures normally require between 20 and 250 ml of water sample to perform the determinations, unless a large number of elements are planned to be analysed or many replicates are required for each experimental unit.

**Table 4:** Drone lifting capacity and amount of water collected using a drone-based methodology during a single flight. The values indicated for each study were obtained using different brand and drone models and different devices or sampling designs. In many cases, the sums were the result of water collected by all available vials per flight and not by a single bottle (for example, for Ore et al., 2015, their sampling mechanism could hold three 20-ml vials, resulting in 60 ml in total)

Drone model	Drone lifting capacity (g)	Amount of water collected (ml) by drone	Amount of water collected/drone lifting capacity ratio	Research group
AscTec Firefly	600	60	0.10	Detweiler et al. (2015), Ore et al. (2015)
Luce Search	5,000	1,000	0.20	Doi et al. (2017)
*Custom-built*	750	130	0.17	Koparan et al., (2018a, 2018b a and b)
LAB645	12,000	330	0.03	Terada et al. (2018)
DJI Phantom 4	500	50	0.10	Benson et al. (2019), Filippi et al. (2021)
DJI Matrice 600	6,000	1,750	0.29	Castendyk et al. (2021)
DJI Matrice 600	6,000	2,000	0.33	Lally et al. (2020), Graham et al. (2022)
*Not mentioned*	5,000	2,000	0.40	Manoharan et al. (2021)
Aurelia X6 Standard	5,000	750	0.15	Hogdson et al. (2022)
DJI Mavic Pro	500	200	0.40	Neto et al. (2022)
Spiri Mu	1,000	250	0.25	Horricks et al. (2022)

As seen in Table 4, only Manoharan et al. (2021), Lally et al. (2020) and Graham et al. (2022) were able to collect a 2-L sample, and in the other reported cases, the amount was considerably smaller. The amount of water that can be collected is not only related to the weight of the drone but also to the lifting capacity, which is the difference between the maximum payload and the weight of the drone with only batteries. Furthermore, the volume of water that can be retrieved (assuming a water density of 1 g/ml) is much less than the drone lifting capacity, with collected water/lifting capacity ratios ranging from 0.4 in Neto et al. (2022) to low values such as 0.03 in Terada et al. (2018). This is because most of the payload capacity is used with the sampling devices, cameras, sensors or floating attachments.

### Limitations of the study and lessons learnt

A limitation of the study lays in the inconsistent results obtained for some variables, particularly at a depth of 3 m, when comparing the two methodologies. Furthermore, the determination of certain variables (such as pH) could have been more accurate if the measurements had been conducted in situ rather than after the delay of transporting the samples to a laboratory.

Additionally, a factor that was not investigated due to the scope of the work, but which could have been implemented, is the attachment of the DJI Zenmuse Gimbal camera, which is included with the purchase of the aircraft. This camera could be useful for sampling using mission planning or for longer-range sampling, allowing for the verification of surface or hydrochemical characteristics of the lake, or even for filming the sampling with greater precision and proximity.

Finally, although sampling in extremely windy and rainy conditions remains a limitation for both boat and drone sampling, models with increasing waterproof capacities are constantly being released to the market. However, the described wide operating range of 70 °C of the DJI Matrice 300 RTK, its high protection against rain and the elevated wind threshold make this device and many of the similar drones offered on the market suitable for safe water sampling for a generous variety of weather conditions.

## Conclusions

Nine out of ten physicochemical variables assessed by multiparameter sonde or analysis of lake water samples showed no statistically significant differences between the boat and drone sampling methodology. pH was the only variable that showed consistently higher values with the drone methodology, the first hypothesis of the chapter being largely fulfilled. Given the instability of the pH, measuring it on site immediately after sampling could help yield more accurate results. Overall, the methods show great consistency, and the drone methodology can adequately replace or complement the traditional boat approach providing reliable results.

The cost-efficiency analysis confirmed that boat-based water sampling takes considerably longer than the drone-assisted methodology. While the latter has higher initial costs, related to the aircraft cost, the faster sampling and the need to employ only one person offset the initial capital expenditure in the medium and long term, if frequent or systematic lake sampling is conducted. Therefore, the drone methodology allows many lake samples in a relatively short time and also with higher levels of safety compared to the boat methodology, which necessarily requires entering the lake and having direct contact with the water.

While our study group prioritised lakes with a surface area of less than 10 ha as a first approach to the topic, the drone water sampling methodology is also suitable for lakes of an order of magnitude larger, such as 50 to 100 ha, a very common scale in Ireland. Despite the larger size of these lakes, drone sampling in the centre of the lake typically does not exceed the visual line of sight, as many lakes are oval-shaped and the centre is close to the shore in some parts of its perimeter, making the methodology more convenient than using a boat. In addition, drones can be useful for large lakes to investigate possible spatial variability in the parameters studied.

Overall, drone-based lake water sampling proved to be a valuable method considering numerous aspects such as consistency of results, cost-efficiency analysis and capabilities of sampling formerly inaccessible lakes. Drone-based water sampling is an alternative that can complement or replace the traditional technique and is particularly useful when the work to be carried out is large-scale, both in terms of time and area. The procedures described in our case study, with corresponding variations according on the specificities of each investigation, are easily replicable for individual, private, university or governmental agencies’ large-scale water sampling campaigns.

## Supplementary Information

Below is the link to the electronic supplementary material.

Supplementary file1 (DOCX 18.8 KB)

## Data Availability

Data will be made available upon reasonable request.
