# Supplementary material for: Efficiency of drone technology for lake water sampling: is it better than traditional boat methodology?
Source: Environ Monit Assess. 2026 May 22;198(6):637. doi: 10.1007/s10661-026-15473-0 (PMC13197280; doi:10.1007/s10661-026-15473-0)
Supplement: Supplementary file 1 — Supplementary file1 (DOCX 18.8 KB) [file 10661_2026_15473_MOESM1_ESM.docx]

Environmental Monitoring and Assessment Journal

***Efficiency of drone technology for lake water sampling: is it better than traditional boat methodology?***

**Authors: Juan Federico Bennett ^1,*^, Brian Rippey ^1^, Richard Douglas ^1^**

*^1^ Ulster University, School of Geography and Environmental Sciences, Coleraine, UK (Cromore Rd, Coleraine BT52 1SA,)*

**Corresponding author: Juan Federico Bennett*

**Further information about the materials and methods of the study**

*UAV and sampling system features*

The DJI Matrice 300 RTK uses simultaneously two identical TB60 batteries to operate (5935 mAh at 52.8 V). The drone is also equipped with a portable BS60 battery station. Despite not having used this function, the batteries are “hot-swappable”, meaning they can be changed one by one without turning off the aircraft. The six drone batteries and the two smart controller batteries are fully charged in approximately 1.5 hours from a normal electrical socket.

The Ingress protection (IP) measures electronic resistance to liquids and due to its airtight manufacturing and protective coating on components, this model was awarded by the Centre Testing International Group Co., Ltd with an IP of 45. That means it is resistant to water projected at it by a 6.3 mm nozzle at 12.5 litres/minute from any direction from a distance of 3 m and to solid objects greater than 1.0 mm in diameter, without compromising internal electronic components.

The YSI EXO 1 sonde operates with two size D alkaline batteries that last approximately 90 days at 20 ℃ at 15-minute logging interval. However, the duration depends not only on water temperature but also on the sensor configuration, batteries brand, and higher measurement frequencies result in significantly shorter durations (YSI, 2024).

*Probe calibration*

To calibrate the YSI EXO 1 dissolved oxygen probe, the oxygen sensor was dried with a clean tissue. The probe was then placed in a calibration container with a little water at the base for fifteen minutes, to saturate the cubicle with water vapor without allowing the cubicle to become sealed or airtight from the outside and allowing the temperature and DO stabilize. Subsequently, the dissolved oxygen option was selected in the calibration menu. The calibration software asks the user to enter atmospheric pressure, which can be measured with another already calibrated probe. After stabilization of the values, the calibration was completed and the values ​​were accepted. This was a simple one-point calibration of water saturated air.

To calibrate the specific conductivity, a small amount of conductivity standard (1413 μS/cm) was poured into the calibration container, ensuring that the vent holes covered the corresponding sensor. The immersed probe was gently rotated to remove bubbles from the cell and it took two minutes for the temperature to stabilize. In the calibration software, the conductivity option and then the specific conductance option were selected, which considers an adjustment by temperature. After stabilization of the values, the calibration was complete, and the values ​​were accepted. This was a simple one-point calibration using a 1413 μS/cm standard.

To calibrate turbidity, a two-point calibration was performed (0 FNU which is high quality water and 124 FNU). First, a small amount of FNU 0 standard was poured in the calibration cup and the probe was immersed, taking care not to trap bubbles in the sensor. In the calibration menu, 0 was entered as the first calibration point and after value stabilization, the point was accepted. The same procedure was conducted for the second point, replacing the 0 FNU standard with the 124 FNU standard and entering 124 FNU in the calibration menu. After stabilization of the values, the entire calibration was accepted.

*Drone flying procedure*

The flights in our study were performed in the manual flight modality. To initiate it after turning on the drone, the “pilot” button has to be clicked in the main menu of the joystick-operated Smart controller and then select “access camera view”. Next, there is a checklist available to check different flight settings before beginning the operation. Afterwards, when the user is finally visualizing the camera and ready to fly, three horizontal dots are available to click in the upper right part of the screen, where the same and other flight settings can be edited, even during the flight. The flight settings are related to items such as maximum altitude, obstacle avoidance, signal lost action or maximum flight distance. The in-flight camera interface also displays live information related to altitude, available satellites for GPS support, battery charge, coordinates and offers zoom and recording functions. The tripod flight mode was selected since it makes the drone move smoother and more gently and, for our sampling purposes, was more convenient than the sport or positioning mode.

In our study, the selected distances for the obstacle avoidance alerts and brakes were only 1 m for horizontal and upward sensing. The study site for the three lakes presented no major obstacles and the whole manual flight route was conducted in a clear area, with no challenging or narrow movements and always flying within visible line of sight. The alert sound of the obstacle avoidance sensors is often disturbing and if we chose to activate a vertical alarm greater than 2 m distance, the noise would have been present during the whole landing, take off and most hover procedures over the lake. The drone was never less than 2 m near the water surface since the connecting rope was 5 m long and the greatest depth measured was 3 m, so the distance was safe and clearly detectable from the shore by the operator.

*Downloading data from the YSI EXO 1 to a laptop*

Kor is the software developed by YSI to interact with the YSI EXO 1 and other models of YSI sondes to visualize, download data and customize all the settings related to the use of the sonde, such as calibrations of the probes (with past records and future reminders), management of sites or selection of available units for the measured variables. Kor has a simple interface consisting in a menu with the functions that can be run and different advanced settings that can be easily edited.

To use that function, the deployment tab in the upper menu must be clicked and the “create template” option must be selected. The template allows to name the study site and choose the logging interval time (in our case one second was chosen). After creating the desired template, the “save and apply template to sonde” button must be clicked. Before the drone takes off with the attached sonde, the “start deployment” option must be selected in the same menu to start recording data. Once the sonde is returned to the shore, the program will show there are no instruments connected as the signal will continue to be lost. After clicking the option” scan for Bluetooth devices” in the main interface, the YSI EXO 1 will be rapidly recognized again. Next, the option “stop deployment” must be clicked in the same submenu and then the “download info from the sensor” must be selected. The program allows to save the file in a spreadsheet format, compatible with Microsoft Excel, with every measured variable corresponding to a different column and every logging corresponding to a different row.

As at the beginning and the end of the deployment period, the recorded values will be from air, it is easy to recognize the exact period where the sonde was submerged in water, as some key variables, such as specific conductivity, will suddenly shift from zero or close to zero to higher values, typical of freshwater lakes or the measured aquatic environment.

Although we did not use it, YSI EXO offers a system that can simplify data collection even when the YSI EXO is far away in the middle of the Lake. This is the EXO GO, a wireless communication device that, through Bluetooth, connects a windows OS device to the YSI EXO sonde, permitting a free hand operation, viewing the evolution of the variables or downloading them while the sonde is submerged (YSI, 2024). For instance, Lally et al. (2020) and Graham et al. (2022) DJI Matrice 600 Pro Hexarotor based water sampling in Irish lakes was conducted with this system.
